# High Burden of Neurodevelopmental Delay among Children Born to Women with Obstructed Labour in Eastern Uganda: A Cohort Study

**DOI:** 10.3390/ijerph20043470

**Published:** 2023-02-16

**Authors:** Martin Chebet, Milton W. Musaba, David Mukunya, Brian Makoko, Agnes Napyo, Ritah Nantale, Proscovia Auma, Ketty Atim, Doreck Nahurira, Seungwon Lee, Dedan Okello, Lawrence Ssegawa, Kieran Bromley, Kathy Burgoine, Grace Ndeezi, James K. Tumwine, Julius Wandabwa, Sarah Kiguli

**Affiliations:** 1 Department of Pediatrics and Child Health, Busitema University, Mbale P.O. Box 1460, Uganda; 2 Department of Global Public Health and Primary Health Care, Centre for International Health, University of Bergen, 5007 Bergen, Norway; 3 Department of Obstetrics and Gynecology, Busitema University, Mbale P.O. Box 1460, Uganda; 4 Department of Community and Public Health, Busitema University, Mbale P.O. Box 1460, Uganda; 5 Department of Research, Nikao Medical Center, Kampala P.O. Box 10005, Uganda; 6 Department of Obstetrics and Gynecology, Mbale Regional Referral Hospital, Mbale P.O. Box 921, Uganda; 7 Department of Neuroscience, University of Pennsylvania, Philadelphia, PA 19104, USA; 8 Department of Research, Sanyu Africa Research Institute, Mbale P.O. Box 2190, Uganda; 9 Research Institute for Primary Care and Health Sciences, School of Medicine, Keele University, Newcastle ST5 5BG, UK; 10 Neonatal Unit, Mbale Regional Referral Hospital, Mbale P.O. Box 921, Uganda; 11 Department of Pediatrics and Child Health, Makerere University, Kampala P.O. Box 7062, Uganda; 12 Department of Pediatrics and Child Health, Kabale University, Kabale P.O. Box 317, Uganda

**Keywords:** neurodevelopmental delay, growth, Eastern-Uganda, thrive, development, nutrition

## Abstract

Over 250 million infants in low and middle-income countries do not fulfill their neurodevelopment potential. In this study, we assessed the incidence and risk factors for neurodevelopmental delay (NDD) among children born following obstructed labor in Eastern Uganda. Between October 2021 and April 2022, we conducted a cohort study of 155 children (aged 25 to 44 months), born at term and assessed their neurodevelopment using the Malawi Developmental Assessment Tool. We assessed the gross motor, fine motor, language and social domains of neurodevelopment. The incidence of neurodevelopmental delay by 25 to 44 months was 67.7% (105/155) (95% CI: 59.8–75.0). Children belonging to the poorest wealth quintile had 83% higher risk of NDD compared to children belonging to the richest quintile (ARR (Adjusted Risk Ratio): 1.83; 95% CI (Confidence Interval): [1.13, 2.94]). Children fed the recommended meal diversity had 25% lower risk of neurodevelopmental delay compared to children who did not (ARR: 0.75; 95% CI: [0.60, 0.94]). Children who were exclusively breastfed for the first 6 months had 27% lower risk of neurodevelopmental delay compared to children who were not (ARR: 0.73; 95% CI: [0.56, 0.96]). We recommend that infants born following obstructed labor undergo neurodevelopmental delay screening.

## 1. Introduction

Over 250 million infants in low and middle-income countries do not fulfill their neurodevelopment potential, which results in poor school performance and intergenerational poverty [1,2]. There is a paucity of epidemiological data on neurodevelopmental disabilities in sub-Saharan Africa [3]. A study in the Mayuge district, Eastern Uganda reported a 13% prevalence of neurodevelopmental disability among premature babies at the age of 12 months as evidenced by gross motor, fine motor, language and social domain challenges [4]. While neonatal complications were key risk factors for neurodevelopmental abnormality, markers of intrapartum complications were not studied [4].

Growing evidence reveals that newborn conditions, such as prematurity, low birth weight, pathological jaundice, infections and intrapartum asphyxia, are risk factors for neurodevelopment disability [4,5,6,7,8]. Intrapartum asphyxia is most commonly caused by obstructed labor, which is characterized by strong and frequent uterine contractions [5]. Obstructed labor impairs blood supply and oxygenation to the placental bed, promoting anaerobic respiration and the accumulation of lactate [9,10]. As a result, hydrogen ions easily cross the placental barrier to reach the fetus, which is known to cause low fetal pH, low Apgar scores, and acidosis in the newborn [11]. Prolonged, uncorrected acidosis (accumulation of H^+^ ions) leads to failure of basic cellular functions, resulting in cellular death [10,12,13].

It is well documented that infants who experience neonatal encephalopathy following intrapartum asphyxia have long-term sequelae including developmental, visual and hearing impairments, cerebral palsy and seizure disorders [14,15]. Data from Uganda show that 29% of infants who survived neonatal encephalopathy had neurodevelopmental impairment at 27–30 months [16]. In many low-resource settings, cord blood gases are not available, meaning that high risk infants are not identified and admitted for observation, and because neonatal seizures can be sub-clinical, it is possible that they go undetected, meaning that prolonged labor alone may be a risk factor for poor childhood outcomes, even without admission to a neonatal unit.

Therefore, in this study we have assessed the incidence and risk factors for neurodevelopmental disability among children born to women with obstructed labor in Eastern Uganda.

## 2. Materials and Methods

### 2.1. Study Design

This was a cohort study among children aged between 25 and 44 months, born following obstructed labor.

### 2.2. Study Setting

The study was conducted in Mbale Regional Referral and Teaching Hospital (MRRH) between October 2021 and April 2022. Mbale Regional Referral hospital is a public tertiary hospital located in Eastern Uganda that serves 14 districts with a catchment population of about 4 million people. It is the main referral center for four district hospitals and 10 health sub-districts in the Elgon sub-region. Approximately 12,000 women deliver at MRRH annually, of whom approximately 500 experience obstructed labour [17].

### 2.3. Study Participants

These were children aged between 25 and 44 months, born to women with obstructed labor. Between July 2018 and September 2019, we conducted a trial to determine the effect of a sodium bicarbonate infusion on blood lactate and maternal and perinatal outcomes among women with obstructed labour in Mbale Regional Referral Hospital. In the randomized controlled trial, we enrolled all eligible patients with obstructed labour, diagnosed by either an obstetrician or a Medical Officer using a definition of the American Association of Obstetricians and Gynaecologists (ACOG). We included full-term, singleton infants in cephalic presentation. We excluded those with medical comorbidities or other obstetric emergencies, such as pre-eclampsia. Trial registration number; PACTR201805003364421 [18]. In this trial, a single intravenous dose of 4.2 g of sodium bicarbonate given in the preoperative period made little or no difference on lactate levels in the baby at birth. Differences were noted in the myometrial and venous lactate levels, which is some evidence of a delayed direct effect [19].

In the current study, we followed up these participants between October 2021 and April 2022 to assess their neurodevelopment.

### 2.4. Sample Size Estimation

The sample size was calculated using Open-Epi (http://www.openepi.com (accessed on 29 September 2020)). We assumed an incidence of 13% neurodevelopmental delay among infants, which was obtained in a study conducted in Mayuge District, Eastern Uganda [4]. We further assumed a precision of 5% and a design effect of 1.0. This gave us a total sample size of 174 participants.

### 2.5. Study Procedures

The mothers and/or caregivers of the participants of the sodium bicarbonate trial were contacted through telephone calls using the contact information that had been captured at the time of enrollment during the baseline Study [19]. Mothers and/or caregivers were invited to bring their children to Mbale regional referral hospital for neurodevelopmental assessment, and informed consent was obtained from the mothers and/or caregivers. Data were collected on the child’s medical history, nutrition, growth, and development. A pediatrician or trained medical officer conducted a detailed physical examination. Weight was measured in duplicate to the nearest 100 g using electronic scales (Seca model 8811021659, Hamburg, Germany). Length was measured in duplicate to the nearest 1 mm with a wooden length board. The Stata package “zscore06” was used to calculate weight for height z-score (WHZ) and height-for-age z-score (HAZ). Mid-upper arm circumference (MUAC) was measured in duplicate to the nearest 1 mm, at the midpoint between the olecranon and the acromion process of the left arm using a standard measuring tape. The overall reliability was substantial with a kappa value of more than 0.75. The key exposure variable was the level of maternal blood lactates categorized as high or low, using a cutoff of 4.8 mmol/L [19].

A food diversity questionnaire was also administered. A minimum acceptable diet was defined as the consumption of four of seven (4/7) food groups and at least four (4) meals a day. To achieve a dietary diversity score, ingredients were categorized into seven food groups, which include grains, roots and tubers; legumes and nuts; dairy products (milk, yoghurt, cheese); flesh foods (meat, fish, poultry and liver/organ meats); eggs; vitamin-A rich fruits and vegetables; other fruits and vegetables [20]. Children who satisfied the minimum acceptable diet were coded as 1 while children who did not were coded as 0. Nutrition assessment was followed by a detailed neurological examination. A trained team of two midwives used the Malawi Developmental Assessment Tool (MDAT) to assess the neurodevelopment of the children [14]. MDAT is a culturally relevant tool that has been created and validated to be used in similar resource-limited settings to measure neurodevelopment delay (NDD) in children from birth to six years in four domains: (1) gross motor, (2) fine motor, and (3) language development through direct observation of the child and (4) social development through questions to the caregiver. The validation tests of the MDAT tool indicate a high sensitivity (97%) and specificity (82%). Each item marked as “pass” or “fail,” and if the child was uncooperative, it was labeled as “don’t know” [21]. The tool’s language and fine motor domains test the child’s cognitive functions, such as problem solving, attention, and pre-academic knowledge of numbers (e.g., asking the child to find an object under a piece of cloth to assess fine motor, problem solving, and memory) [22]. When a child failed six consecutive items within a domain, the assessor moved on to the next domain. MDAT assessments were conducted by two midwives who had been trained by a team consisting of a pediatric neurologist, two pediatricians with extensive experience in assessing neurodevelopment, and a colleague that had previously conducted neurodevelopment assessment in the Iganga-Mayuge DSS as a part of her PhD [23]. An experienced pediatrician supervised the research team. In the primary Sodium Bicarbonate Study, socio-demographic information as well as maternal and cord blood lactate levels were collected.

### 2.6. Outcome Variables

The outcome variable was occurrence of neurodevelopmental delay among children born to women with obstructed labour. The MDAT has four domains: gross motor, fine motor, language, and social development. A child was determined to have failed if he/she failed more than two parameters of the age-based developmental expectations. The four domains were used to compute a score that was used to classify the outcome into children with or without neurodevelopment delay.

### 2.7. Independent Variables

The independent variables included the sociodemographic characteristics of the mothers and child, clinical characteristics, maternal and cord lactate levels at birth, and nutritional status of the child.

At the time of the sodium bicarbonate trail, maternal lactate and amblical cord lactate were taken for each participant. This cord lactate level was retrieved from the data and was as one of the independent variables for this study.

### 2.8. Statistical Analysis

Data were entered using Open Data Kit and then exported into Stata Software Version 14.0 (StataCorp; College Station, TX, USA) for analysis. We entered the neurodevelopment score from the MDAT assessment into the already existing dataset in Stata Software Version 14.0 (Stata Corp; College Station, TX, USA) to make a complete dataset of exposures and the outcome.

Categorical variables such as neurodevelpmental status, sex, maternal educational status, wealth quintile, cord lactate level and others were summarized into frequencies with percentages and continuous variables such as age into means or medians with corresponding standard deviations and IQR, respectively. Poor neurodevelopment was defined as a score of less than −2 development for age Z scores on the full model from the original Malawi population that was used to validate the tool (https://kieran-bromley.shinyapps.io/mdat_scoring_shiny/ (accessed on 15 August 2022)). We conducted bivariable and multivariable analysis using a generalized linear model for the Poisson family with a log link to assess the strength of association, using risk ratios between exposures and the incidence of neurodevelopmental delay.

## 3. Results

### 3.1. Study Profile

**Figure 1:**
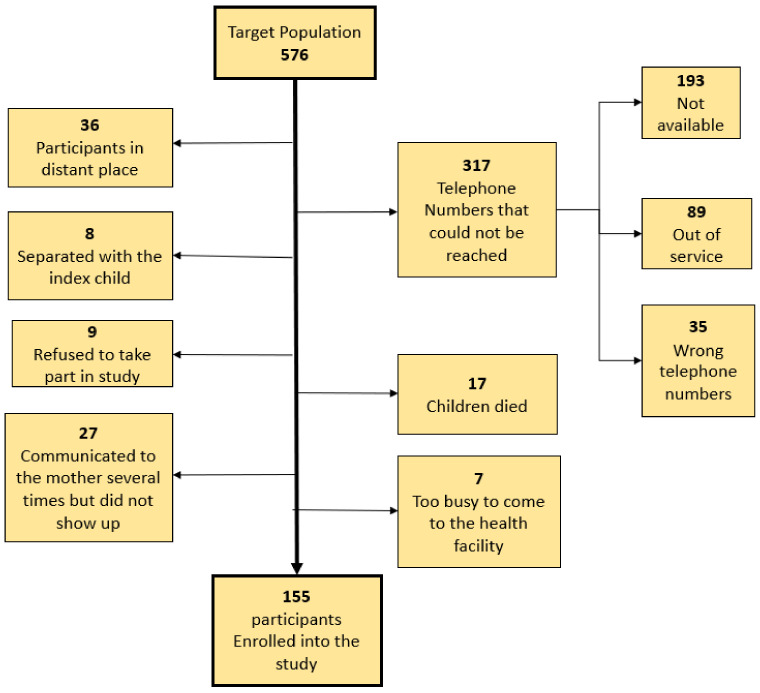
The study profile.

In the clincial trial, we enrolled 576 children. Out of these, we were able to enroll 155 into the current study. We failed to reach 317 participants on the phone because the telephone contacts were not reachable. The rest of the reasons for not enrolling other eligible children into this study are shown in the study profile, (Figure 1).

### 3.2. Participant Characteristics

Table 1 summarizes the participant characteristics of children in this cohort. We enrolled 155 (Figure 1) children with a mean age of 33.8 months (ranged 25 to 44 months), standard deviation (SD) of 4.7 months. The educational status for most mothers at the time of birth was either primary or secondary school (59/155 and 75/155, respectively). The mean (SD) maternal age at childbirth was 25.7 (6.3). Most of the participants were male (101/155, 65.2%). Almost all births were by cesarean section (146/155) and only 23 neonates were admitted to the neonatal unit (12 of these developed NDD). The mean umbilical lactate level at birth among the study participants was 10.1 with a standard deviation (SD) of 6.1.

### 3.3. Neurodevelopmental Delay among Children Born following Obstructed Labor in Eastern Uganda

Overall, 105/155 (67.7%: 95% CI (Confidence Interval): 59.8–75.0) children had neurodevelopmental delay. Across the domains, delay was observed as follows: gross motor domain, 129/155 (83.2%: 95% CI: 76.4–88.7), fine motor domain, 65/155 (41.9%: 95% CI: 34.1–50.1), language domain, 136/155 (87.7%: 95% CI: 81.5–92.5), social domain, 77/155 (49.7%: 95% CI: 41.6–57.8) (Table 2).

#### Risk Factors of Neurodevelopmental Delay among Children Born following Obstructed Labor in Eastern Uganda

Table 3 summarizes factors associated with neurodevelopmental delay (NDD). Children belonging to the poorest quintile had 83% higher risk of NDD compared to children belonging to the richest quintile (ARR (Adjusted Risk Ratio): 1.83; 95% CI (Confidence Interval): [1.13, 2.94]). Children who had the recommended meal diversity (eating from 4 out of 7 recommended food groups) had a 25% lower risk of NDD compared to children who did not (ARR: 0.75; 95% CI: [0.60, 0.94]). Children who were exclusively breastfed in the first 6 months had a 27% lower risk of NDD compared to children who were not (ARR: 0.73; 95% CI: [0.56, 0.96]). There was no association between umbilical cord lactate levels and neurodevelopmental delay.

## 4. Discussion

In this cohort study, we followed up children born following obstructed labor in a regional referral hospital in Eastern Uganda to determine the incidence of and risk factors for neurodevelopmental delay. We found a very high incidence of neurodevelopmental delay. Children that belonged to the highest income quintile, satisfied meal diversity recommendations, and were exclusively breastfed for their first six months had a lower risk of neurodevelopmental delay.

Our finding of a very high incidence of neurodevelopmental delay was surprising but could be explained by the high morbidity associated with obstructed labour. Over 90% of the 26,000 perinatal deaths audited and reported to the Ministry of Health in Uganda annually are attributed to intrapartum birth asphyxia. Obstructed labour is a known risk factor for neonatal encephalopathy, which is associated with neurological dysfunction and a risk of long-term sequelae, including developmental, visual and hearing impairments, cerebral palsy and seizure disorders [14,15]. Our study demonstrated an unexpectedly high incidence of NDI (67.7%), more than double that seen by Cally et al. in a cohort of Ugandan infants who were admitted for and survived neonatal encephalopathy (29%) [16]. This is possibly due to the different tools that were used and that our cohort was seen at an older age allowing a more accurate evaluation of neurodevelopment. There is also a difference in the populations studied because the children in our study were born following obstructed labor while those in Cally’s study had encephalopathy due to different perinatal conditions. Our findings are also higher than the findings of Namazzi et al. who studied the prevalence of neurodevelopmental delay among all infants born in a district hopsital in Eastern Uganda. The difference in the findings may be because the populations studied were different.

All of the children who underwent assessments in the study had experience obstructed labour, and almost 80% of them had an elevated lactic acid level. Only 15% were admitted to the neonatal unit with signs of neonatal encephalopathy, and yet this group of infants seems to be at incredibly high risk of NDI, made worse by poor nutritional intake and low socio-economic status. In HICs, infants with abnormal blood gases would be admitted for observation and management in a neonatal unit. These infants would undergo Cerebral Function Monitoring to monitor for sub-clinical seizure activity and treated as required. In addition, cord blood gas analysis is used to guide therapeutic hypothermia, which is known to reduce the incidence of NDD and cerebral palsy in HICs. In many low-resource settings, cord blood gases are not available, meaning that high risk infants are not necessarily identified and admitted for observation, and because neonatal seizures can be sub-clinical, it is possible that they go undetected, meaning that prolonged labour alone may be a risk factor for poor childhood outcomes, even without admission to a neonatal unit. In reality, infants that are admitted to neonatal units with neonatal encephalopathy in LICs are already severely encephalopathic, often with clinically detectable seizures evident within 6 h of delivery. It is highly possible that the infants in our study had experienced significant hypoxic ischaemic damage that although was not clinically detectable, would perhaps have warranted treatment in a high-resource setting. We did not find similar studies for comparison except Namazzi et al. who reported a 13% prevalence of neurodevelopmental delay among premature babies at the age of 12 months post-delivery in Mayuge district [23]. Although the two studies used the same assessment tool (MDAT), the observed differences may be attributed to differences in the obstetric and intra-partum exposures. This finding has important clinical and policy implications as it emphasizes closer follow-up and careful assessment of children born to mothers with obstructed labor, to help them reach their full neurological potential. In addition, this study suggests that efforts to prevent obstructed labour, a known risk factor for intra-partum asphyxia, through improved intra-partum care, can minimize neurodevelopmental delay. Lastly, the role of additional investigations and treatment in these infants who would not be traditionally admitted to a neonatal unit in a low-resource setting deserves urgent attention.

As expected, exclusive breast-feeding in the first six months was associated with a lower risk of neurodevelopmental delay. This is consistent with findings from earlier studies conducted in diverse settings [24,25]. This positive association could be due to the presence of long-chain polyunsaturated fatty acids, such as arachidonic acid and docosahexaenoic acid, in breast milk that are important for brain development [26]. In addition, breastfeeding helps mothers to bond with their child, which positively contributes to child development [27,28]. A comparable study from Western Kenya that used a different assessment tool and follow-up age of children demonstrated that exclusive breastfeeding for 3–6 months is positively associated with childhood development [29]. However, Tumwine et al. [30] reported a negative association between exclusive breastfeeding and cognitive function from the PROMISE EBF cluster randomized trial conducted in Uganda and Burkina Faso. One possible explanation for this variation may be due to differences in the tool used to assess cognitive function. These findings have important clinical and public health implications for child health, growth, and development as it highlights the importance of exclusive breastfeeding for child survival and healthy neurological development.

We also found that children born to women belonging to the highest income quintile and those that had followed the recommended meal diversity were less likely to have neurodevelopmental delay. While not surprising, this may be a product of advantaged social position, education, or parenting style, which translates into mothers having more time off work to exclusively breastfeed their newborns and also be in a more convenient position to provide adequate meals for their families.

### Strength and Limitations

Our study is among the first to assess neurodevelopmental delay among children born to women diagnosed with obstructed labour, which is a major cause of neonatal morbidity and mortality in low-resource settings. One weakness was a low response rate, which makes our study prone to selection bias. The direction of bias could have been either way as those who did not come for follow up could have been healthier or sicker. We also did not conduct genetic tests for genetic abdnormalities and did not assess corpus callosum abnormalities, epilepsy, and isolated electroencephalogram abnormalities. The study was not originally designed to assess neurodevelopment in infants so we did not prospectively capture some exposures such us infections over the follow-up period.

## 5. Conclusions

Two thirds of children born to women with obstructed labour, most of whom had an associated lactic acidosis but were not admitted to the neonatal unit, had a neurodevelopmental delay. Further biochemical, radiological and electroencephalographical descriptions of this population are urgently needed in order to understand how the management of these infants could be improved in the perinatal period. In addition, promotion of exclusive breastfeeding and access to adequate meals for all should be promoted to reduce the risk of NDD. We recommend similar studies be conducted in different populations of children in Uganda. Lastly, we must continue to advocate for quality antenatal and perinatal care to prevent perinatal asphyxia.

## Figures and Tables

**Table 1:** Characteristics of children born to women with obstructed labor in eastern Uganda.

Variable	Normal (*n* = 50)	NDD (*n* = 105)	Total (155)
Child age (months)	33.70 (4.8)	33.91 (4.7)	33.85 (4.7)
Child head circumference (cm)	49.25 (7.3)	49.69 (6.5)	49.55 (6.8)
Umbilical arterial lactate (mmol/L)			
<4.8	10 (20.0)	24 (22.8)	34 (21.9)
4.8–10	22 (44.0)	47 (44.7)	69 (44.5)
>10	16 (32.0)	33 (31.4)	49 (31.6)
Mother’s education status at child birth			
None	0 (0.0)	2 (1.9)	2 (1.3)
Primary	16 (32.0)	43 (40.9)	59 (38.1)
Secondary	26 (52.0)	49 (46.7)	75 (48.4)
Tertiary	8 (16.0)	10 (9.5)	18 (11.6)
Wealth index			
Q1 (Lowest income)	4 (8.0)	27 (25.7)	31 (20.0)
Q2	8 (16.0)	22 (20.9)	30 (19.4)
Q3	7 (14.0)	23 (21.9)	30 (19.4)
Q4	13 (26.0)	17 (16.2)	30 (19.4)
Q5 (Highest income)	18 (36.0)	12 (11.4)	30 (19.4)
Child sex			
Female	15 (30.0)	35 (33.3)	50 (32.3)
Male	33 (66.0)	67 (63.8)	100 (64.5)
Stunting			
No	43 (86.0)	83 (79.1)	126 (81.3)
Yes	7 (14.0)	22 (20.9)	29 (18.7)
Child’s HIV Status			
Negative	46 (92.0)	93 (88.6)	139 (89.7)
Positive	4 (8.0)	12 (11.4)	16 (10.3)
Maternal Age at Childbirth (years)			
<20	12 (24.0)	15 (14.3)	27 (17.4)
20–30	27 (54.0)	71 (67.6)	98 (63.2)
>30	11 (22.0)	18 (17.1)	29 (18.7)
Child’s diet meets minimum food diversity			
No	2 (4.0)	21 (20.0)	23 (14.8)
Yes	48 (96.0)	84 (80.0)	132 (85.2)
Number of meals per day			
<4 meals a day	13 (26.0)	32 (30.5)	45 (29.0)
4 and above meals	37 (74.0)	69 (65.7)	106 (68.4)
Exclusively breastfed up to 6 months			
No	20 (40.0)	75 (71.4)	95 (61.3)
Yes	30 (60.0)	30 (28.6)	60 (38.7)
Hospitalized with malaria in previous year			
No	44 (88.0)	89 (84.8)	133 (85.8)
Yes	6 (12.0)	16 (15.2)	22 (14.2)

**Table 2:** Neurodevelopmental delay among children born following obstructed labor in Eastern Uganda.

	Total Population		Umbilical Arterial Lactate ≤ 4.8 (*n* = 34)		Umbilical Arterial Lactate > 4.8 (*n* = 118)	
	DAZ Mean (SD)	Delay (*n*, %)	DAZ Mean (SD)	Delay (*n*, %)	DAZ Mean (SD)	Delay (*n*, %)
Overall	−4.4 (4.0)	105 (67.7)	−4.3 (3.8)	24 (70.6)	−4.5 (4.1)	80 (67.8)
Gross Motor	−4.9 (2.3)	129 (83.2)	−5.1 (2.3)	29 (85.3)	−4.9 (2.3)	98 (83.1)
Fine Motor	−3.5 (5.2)	65 (41.9)	−3.7 (5.4)	15 (44.1)	−3.5 (5.2)	50 (42.4)
Language	−4.0 (2.4)	136 (87.7)	−3.8 (2.6)	28 (82.4)	−4.1 (2.3)	106 (89.8)
Social	−2.9 (3.0)	77 (49.7)	−3.0 (3.7)	17 (50.0)	−2.9 (2.8)	59 (50.0)

DAZ: Development-for-Age Z-score.

**Table 3:** Risk factors of neurodevelopmental delay among children born following obstructed labor in Eastern Uganda.

Variable	CRR * [95% CI]	*p*-Value	ARR ** [95% CI]	*p*-Value
Umbilical cord arterial lactate (mmol/L)				
<4.8	1		1	
4.8–10	0.96 [0.74, 1.27]	0.797	0.98 [0.73, 1.31]	0.891
10–25	0.95 [0.71, 1.28]	0.753	0.92 [0.68, 1.24]	0.589
Maternal Education Status				
None/Primary	1		1	
Secondary	0.89 [0.71, 1.11]	0.287	0.96 [0.76, 1.22]	0.746
Tertiary	0.75 [0.48, 1.17]	0.207	1.00 [0.66, 1.51]	0.995
Maternal age				
<30 years	1		1	
31–48 years	0.90 [0.66, 1.23]	0.514	0.86 [0.64, 1.15]	0.31
Wealth index				
Q1 (Lowest income)	2.18 [1.37, 3.45]	0.001	1.83 [1.13, 2.94]	0.014
Q2	1.83 [1.12, 2.99]	0.015	1.52 [0.93, 2.50]	0.098
Q3	1.92 [1.18, 3.10]	0.008	1.68 [1.02, 2.75]	0.041
Q4	1.42 [0.83, 2.43]	0.206	1.29 [0.76, 2.19]	0.349
Q5 (Highest income)	1		1	
Sex				
Female	1		1	
Male	0.96 [0.76, 1.20]	0.707	1.00 [0.79, 1.26]	0.969
Child average head circumference (cm)	1.00 [0.99, 1.02]	0.691	1.01 [0.99, 1.02]	0.43
Stunting				
No	1		1	
Yes	1.15 [0.90, 1.47]	0.252	1.26 [0.97, 1.65]	0.085
HIV Status				
Negative	1		1	
Positive	1.12 [0.82, 1.52]	0.466	1.26 [0.92, 1.75]	0.154
Minimum food diversity				
No	1		1	
Yes	0.70 [0.58, 0.84]	<0.001	0.75 [0.60, 0.94]	0.014
Minimum Meal frequency per day				
<4 meals in a day	1		1	
4 and above meals	0.92 [0.72, 1.16]	0.458	1.07 [0.85, 1.35]	0.577
Exclusive breastfeeding up to 6 months				
No	1		1	
Yes	0.63 [0.48, 0.83]	0.001	0.73 [0.56, 0.96]	0.025
Malaria hospitalization in the past year				
No	1		1	
Yes	1.09 [0.82, 1.44]	0.565	1.26 [0.94, 1.70]	0.12

CRR *: Crude Risk Ratio ARR **: Adjusted Risk Ratio.

## Data Availability

The datasets used and/or analyzed during the current study are available from the corresponding author.
